# CellProfiler Tracer: exploring and validating high-throughput, time-lapse microscopy image data

**DOI:** 10.1186/s12859-015-0759-x

**Published:** 2015-11-04

**Authors:** Mark-Anthony Bray, Anne E. Carpenter

**Affiliations:** Broad Institute of MIT and Harvard, 415 Main St, Cambridge, MA 02142 USA

**Keywords:** CellProfiler, Time lapse, Quality assessment, Fluorescence microscopy, Image analysis, Data visualization, Data exploration

## Abstract

**Background:**

Time-lapse analysis of cellular images is an important and growing need in biology. Algorithms for cell tracking are widely available; what researchers have been missing is a single open-source software package to visualize standard tracking output (from software like CellProfiler) in a way that allows convenient assessment of track quality, especially for researchers tuning tracking parameters for high-content time-lapse experiments. This makes quality assessment and algorithm adjustment a substantial challenge, particularly when dealing with hundreds of time-lapse movies collected in a high-throughput manner.

**Results:**

We present CellProfiler Tracer, a free and open-source tool that complements the object tracking functionality of the CellProfiler biological image analysis package. Tracer allows multi-parametric morphological data to be visualized on object tracks, providing visualizations that have already been validated within the scientific community for time-lapse experiments, and combining them with simple graph-based measures for highlighting possible tracking artifacts.

**Conclusions:**

CellProfiler Tracer is a useful, free tool for inspection and quality control of object tracking data, available from http://www.cellprofiler.org/tracer/.

**Electronic supplementary material:**

The online version of this article (doi:10.1186/s12859-015-0759-x) contains supplementary material, which is available to authorized users.

## Background

Time-lapse assays probe biological questions that can only be investigated by observing the dynamic behavior of organisms, cells, organelles, or molecular assemblies over time [1]. The combination of automated imaging and large-scale, high-content, live-cell experiments is capable of delivering large amounts of data in very little time [2]. However, time-lapse imaging is acutely susceptible to many artifacts that negatively affect the proper identification and tracking of cells; the appearance of such anomalies in a single frame can ruin an entire time series. Thus, image and image analysis quality requirements for time-lapse microscopy are more stringent and, due to the volume of data, automated quality control is more necessary.

Interfaces for review or correction of time-lapse data are sometimes provided in customizable open-source software but are usually manual in nature, requiring visual inspection to detect aberrations [3, 4]. Alternately, commercial software may present such functionality within a polished interface (e.g., Imaris by Bitplane, Volocity by Perkin-Elmer, MetaMorph by Molecular Devices), but such packages are not open-source, precluding access to or adjustment of features and underlying algorithms. We saw a need for a tool that would link cell images themselves directly to their morphological measurements within a tracking assessment tool; without this, a valuable opportunity is missed for the researcher to visually assess important changes in cell morphology and cellular context that accompany particular tracking results.

The CellProfiler biological image analysis package is widely used for collecting an extensive suite of morphological, intensity, and textural features for cells and organisms in high-content screens [5, 6]. Moreover, it includes modular cell-tracking capabilities for time-lapse assays, such as the linear assignment problem (LAP) approach [7], which provides robust tracking by closing temporal gaps and capturing object merges and splits. CellProfiler is one of the few options for conveniently combining the need for robust cellular identification and the ability to process large numbers of time-lapse movies [8–10]. However, configuring tracking algorithm parameters has been tedious without a tool to readily assess track quality. We present CellProfiler Tracer to enable the visualization of the rich set of cellular features characteristic of high-content time-lapse assays, as well as to provide measures for assessing track quality.

## Implementation

**Fig. 1:**
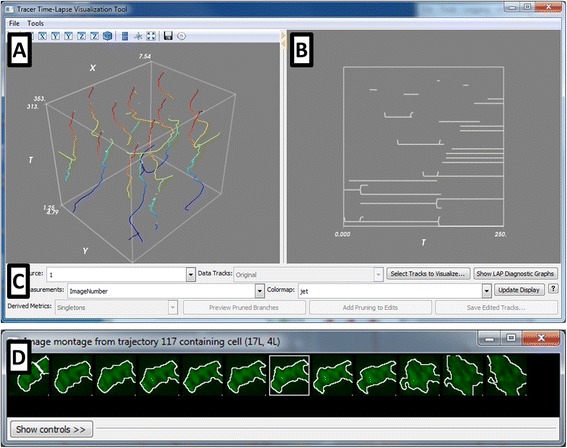
The CellProfiler Tracer interface. The user interface is divided into the (**a**) XYT panel, showing the object trajectories in (x,y,t) coordinates, color-coded here by the frame number; the trajectories can be color-coded to be any cell measurement of interest; (**b**) the lineage tree panel, highlighting the ancestor/progeny relationships corresponding to the trajectories in (**a**), and (**c**) the control panel containing various display tools. Other visualizations include (**d**) synchrograms of selected cells, as well as heatmaps (shown in Fig. 2)
- **Lineage tree** (Fig. 1b): *Lineage trees* omit the positional information of XYT plots to display relationships between cell descendants over time; such graphs are commonly used for developmental mechanism and cell cycle progression studies [4, 16–19]. Each cell at a given timepoint is represented as a node, connected by edges to the tracked predecessors and successors. As with the XYT plot, the nodes can be color-coded according to the desired per-cell image feature. Errors in object segmentation often appear and disappear within a few frames, which results in an object split followed by a re-merge (or vice versa). This topological distortion is most obvious in the lineage display and is used to call attention to the need for adjustment to the image analysis parameters.
- **Synchrograms** (Fig. 1d): A *synchrogram* is a sequence of images of an individual cell over time. This visualization helps track the progression of sub-cellular processes [20]. The selected cell is centered in each frame to remove motion as a visual degree of freedom; this also makes frame-to-frame tracking errors immediately apparent. This tool allows for follow-up on suspect trajectories or lineages identified with the other tools, using a simple point-and-click interface linked to the other plots.
- **Heatmaps** (Fig. 2): *Heatmaps* are used to represent numerical data graphically as a colored two-dimensional matrix. Tracer can average each per-object feature across all trajectories at each timepoint and display a heatmap of the result. This display provides a simple means for the user to visually evaluate the data for significant trends in the cell population, even if the time-lapse data is not temporally synchronous. This can be helpful for quality control purposes (e.g., a given timepoint was transiently out of focus) or perhaps relevant to the phenotype under investigation (e.g., observing trends in response to drug treatment).

CellProfiler Tracer is implemented as part of the CellProfiler Analyst software package [11] (available from http://www.cellprofiler.org). Although most seamlessly used with data from CellProfiler, the Tracer software is intended as a visualization and quality assessment tool compatible with high-content object tracking data derived from any two-dimensional time-lapse image sets, which most commonly involve fluorescence or brightfield microscopy. Thus, Tracer is not itself a tracking algorithm, nor a general-purpose image visualization tool, nor a tool for manual track editing, but may be used in conjunction with other software for those purposes [12, 13]. We used CellProfiler Analyst as the foundation for Tracer, as CellProfiler Analyst was designed for visualizing large, multi-parametric data sets, with the ability to create various plots of cellular features. It emphasizes linking the plotted data to the originating image for visual inspection and improved biological interpretation. To create Tracer, we added visualizations to CellProfiler Analyst that are specific to time-lapse tracking data and that have already been proven useful in the scientific community but have not as yet existed in a single freely-available and open-source software package, namely the following:

- **XYT plot** (Fig. 1a): An *XYT plot* is a 3-D plot of the cell centroid versus time and is useful as a straightforward means of visualizing discrete cellular trajectories [14, 15]. Tracer can color-code the trajectories based on a selected per-cell feature, so that variations in the trajectory color reflect the size, shape, intensity, or other high-content, multi-channel features collected during the experiment. The plot can be rotated using the mouse to view the trajectories from any angle.

**Fig. 2:**
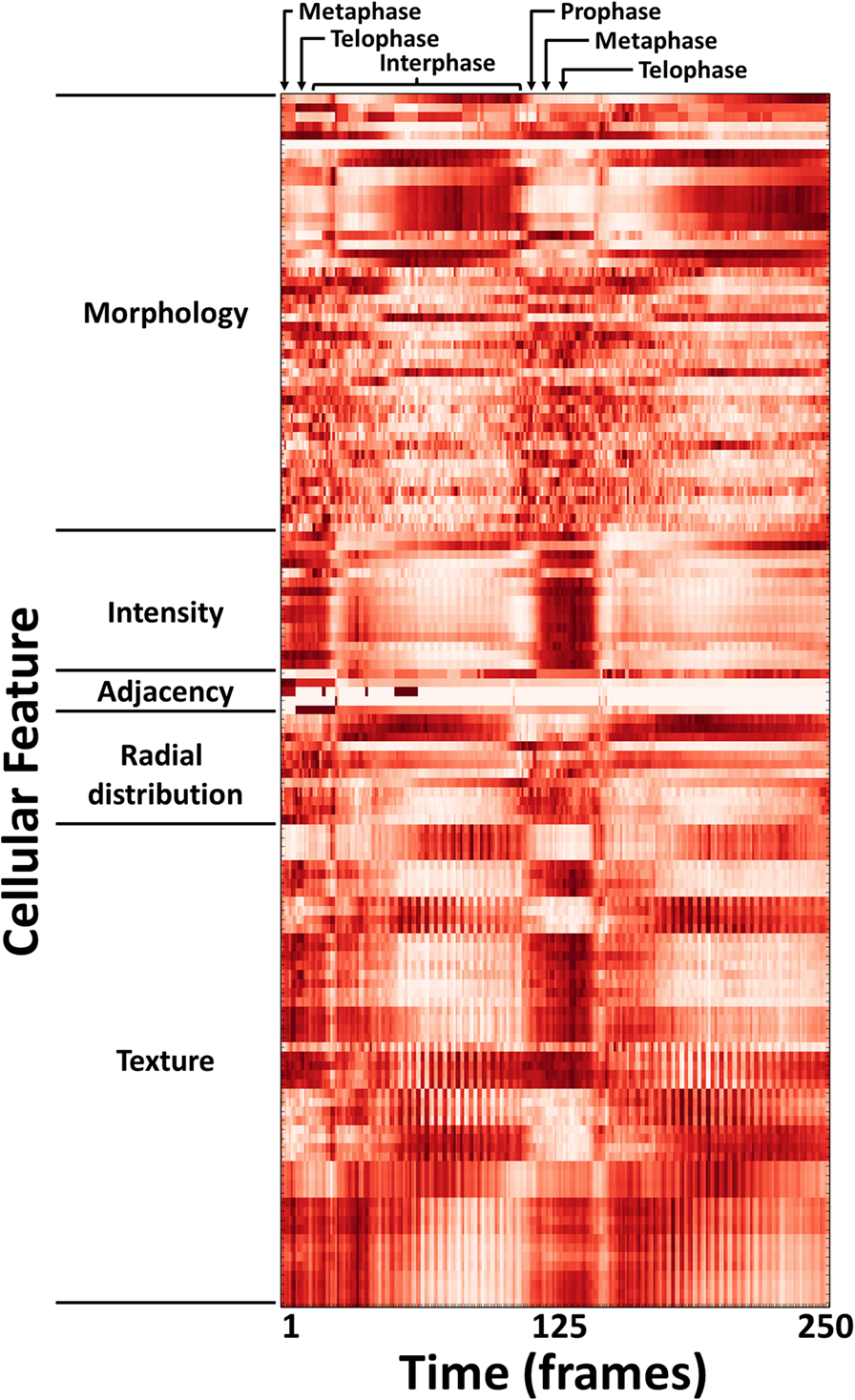
Heatmap of high-content cellular time-lapse measurements. The per-nucleus measurements from a *Drosophila* time-lapse movie are averaged over all nuclei for each timepoint; the measurements were collected by CellProfiler software. Feature values were normalized from 0 to 1 for visualization purposes. Feature names were omitted for conciseness but are provided in the Tracer display; the features shown are listed in order in the Additional file 3: Table S1, and are further described in the CellProfiler documentation

A control panel (Fig. 1c) is used to adjust various aspects of the display panels. Other visualization features, such as plots and trajectory selections, are provided via a context menu available on the display panels (Additional file 1: Figure S1). In addition, diagnostics related to the LAP tracking method [21] and other quality metrics (described below) may also be displayed as an aid to tracking optimization (Additional file 1: Figure S2).

Tracer is designed to process and explore any MySQL- or SQLite-based database of image-based screening data structured according to the following simple schema:

- An image table where each row corresponds to an image acquired at a unique timepoint and field of view and the columns contain the image data (e.g., the name of the treatment condition, the path to and filename of the original image, etc.). A requirement for this table is an image index, given as a column of integers referencing each site (i.e., field of view) acquired.
- An object table in which each row represents an object (e.g., cells) from a given image and the columns contain the collected object measurements (e.g., area of the cell, intensity of DNA stain in the nucleus, location of the cell in the original image). Required for this table is an image index as described above, as well as an object index given as a column of integers referencing each object identified in an image. An (x,y) location for each object is also required (e.g., the cell centroid) as columns in the table; this permits limited 3D + *t* functionality if a 2D centroid of each 3D object is provided rather than an entire 3D segmentation, e.g., by using a maximum projection into the XY plane or a particular Z-slice.
- In addition, an object relationship table is also required. Each row corresponds to the image and object index for a given object and that of its “parent”, i.e., tracked predecessor.

The above data tables can be automatically generated by CellProfiler using its ExportToDatabase and ExportToSpreadsheet modules; example files are provided at http://www.cellprofiler.org/tracer/. However, these schema are intended to be simple enough that a third-party software package (e.g., MATLAB, another object tracking package) can easily format its data accordingly. Using this format, a trajectory (defined as the frame-to-frame path followed by an object over time) can be captured by following the ancestor-progeny mapping for an object. The relationship table captures both one-to-many and many-to-one object mappings created by splits and merges, as well as temporal gaps produced by transient object disappearances.

The primary challenge in handling aberrations in time-lapse data is discriminating between natural biological behaviors versus analysis artifacts (e.g., cell division during mitosis versus improper cell splitting due to mis-segmentation). To address this issue, Tracer allows the user to assess the quality of object trajectories by treating the trajectories as a network graph and highlighting possible aberrations in the graph connectivity. The use of graph-based methods to analyze and resolve defects in time-lapse tracking data has been explored previously [16, 22–25]; here, we use Tracer to simply bring attention to aspects of the network that may represent possible mistakes in cellular segmentation and tracking.

## Results and discussion

We demonstrate the data analysis and quality assessment features of CellProfiler Tracer using two time-lapse movies that reveal the dynamics of nuclear division: a *Drosophila* blastoderm embryo with GFP-histone marking the nuclear DNA (Foe lab, University of Washington, unpublished data used with permission) and MCF-7 nuclei tagged with NLS-mCerulean fusion protein (Ramaswamy lab, Massachusetts General Hospital Cancer Center, unpublished data used with permission); further details on these data and permissions for use are included in the Additional file 2. We used CellProfiler to identify the nuclei, track them over time, and measure over 130 features of area, shape, intensity and texture (see the Additional file 3: Table S1 for the full list of cellular features); the image data and CellProfiler pipelines are available from http://www.cellprofiler.org/tracer/. In the case of the *Drosophila* embryos, the nuclei proceed through the cell cycle in synchrony, due to their sharing a common cytoplasm. From the heatmap shown in Fig. 2, one can see that the rich set of features derived from GFP-histone expression at each time point of the movie could be used to fingerprint nuclei at particular phases of the cell cycle. Similarly, any of the features collected can be visualized on the XYT and lineage panels by selecting the desired measurement for color-coding the object tracks; Additional file 1: Figure S3 shows examples for the two data sets. Hypotheses about cell behavior can be generated and tested using this view.

**Fig. 3:**
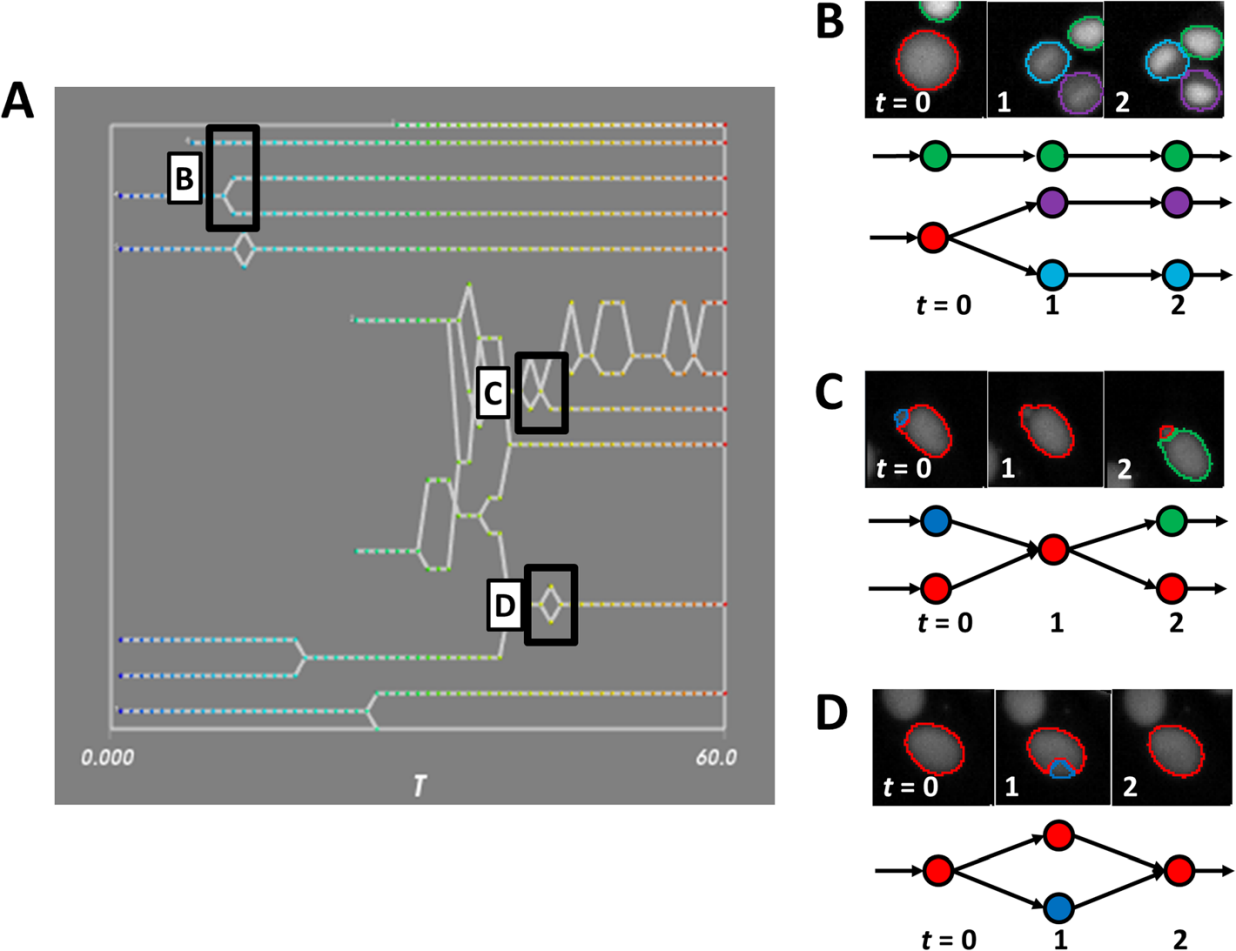
Schematics of tracking errors. **a** An inset of the lineage panel for a movie of MCF-7 cells, with various tracking topologies highlighted. **b-d** Tracking errors are reflected in synchrograms of MCF-7 nuclei (top panel) and graph topologies (bottom panel) with color indicating the unique object label. **b** Typical graphs with no tracking errors. **c** Mis-segmentation of neighboring objects produces transient merging and erroneous object creation. **d** A brief mis-segmentation of an object results in a transient (and incorrect) split

Cells typically exhibit a limited range of dynamic behavior, and hence the resultant network graphs are expected to assume only certain topologies, as shown in a movie of MCF-7 nuclei (Fig. 3a). Therefore, deviations from expected topologies (Fig. 3b) may indicate that something is amiss in the tracking. The user can select to display three different graph deviations in the Tracer interface: loops, crossings, and singletons; all of these are evaluated when the data is first loaded. For example, transient split/merge ("loops") or merge/split ("crossings") events are unlikely to occur in typical biological settings and may indicate an object mis-segmentation (Fig. 3c, d). Likewise, a very short trajectory ("singleton", although the precise number of frames can be selected by the researcher) may correspond to a spurious object detection. For singletons, the user can produce a display showing the distribution of track lengths and a chart listing the total number of tracks and the median, 10th and 90th percentiles of the track lengths (Additional file 1: Figure S4).

CellProfiler Tracer examines the tracking data for these deviations and highlights suspect nodes by color-coding them in the display panels. In the case of singletons, these nodes may be removed to create a new network graph saved with the original data for retrieval across Tracer sessions. It should be emphasized that the highlighted nodes are intended to call attention to possible object segmentation or tracking problems; the absence of suspect nodes in a particular dataset does not preclude other errors. However, a large number of highlighted nodes would indicate the need for further optimization of the original cell segmentation settings. While other tracking interfaces include manual or semi-supervised segmentation editing [4, 26], this functionality is currently outside the scope of Tracer. This is because the tool aims to support the completely automated analysis of thousands of time-lapse image sets, for which manual intervention is impossible and some amount of automated error must be tolerated. Thus, the tool is designed to assist researchers in selecting appropriate segmentation and tracking parameters in other software (like CellProfiler), such that the resulting data files are as high quality as possible. If feasible and necessary (e.g., for small-scale experiments), manual editing of individual trajectories can be carried out using other software, after Tracer has assisted in optimizing the automated segmentation and tracking parameters.

To illustrate the use of CellProfiler Tracer for optimizing parameters, we tracked nuclei in the MCF-7 time-lapse data set using CellProfiler; for this movie, the default settings for its LAP tracking method yielded substantial errors (Additional file 1: Figure S5). Anecdotally, researchers have reported that the lack of visual feedback makes adjusting the algorithm’s many parameters extremely challenging. Using Tracer to visualize and assess the resulting tracks, the tracking settings in CellProfiler were then changed accordingly and the nuclei re-analyzed to produce revised tracks. This procedure was iteratively repeated until the final tracks reflected the actual temporal behavior of the nuclei; these optimized settings were then confirmed in Tracer to reproduce the expected tracking behavior for a different MCF-7 data set (Additional file 1: Figure S6). It bears noting that while only the lineage panel is shown in Additional file 1: Figure S5 for brevity, all three visualization tools were employed for optimization. While this one particular movie could have been analyzed more quickly using a tool for manual correction; the value in using Tracer is to optimize automated settings on subsets of time-lapse data, so that they can be applied to hundreds of thousands of data sets, for example, using CellProfiler’s high-throughput interface.

## Conclusions

As cellular tracking matures and the size of microscopy data sets continues to increase, progress in validating tracking quality will make powerful time-lapse experiments on larger data sets feasible [26–29]. CellProfiler Tracer is a tool that augments the cell-tracking functionality of the CellProfiler biological image analysis package by visualizing multi-parametric time-lapse data. The software incorporates graph-based assessment of tracking quality, and makes it easy to produce and interact with XYT plots, lineage trees, synchrograms and heatmaps — visualizations that have proven useful but have not as yet existed in a single freely-available and open-source software package.

## Availability and requirements

- **Project name:** CellProfiler Tracer
- **Project home page:** http://www.cellprofiler.org/tracer/ (installer), https://github.com/CellProfiler/CellProfiler-Analyst/tree/cellprofiler-tracer (source code)
- **Operating systems:** Windows, 64-bit
- **Programming language:** Python
- **Other requirements:** The Tracer source code for CellProfiler Analyst requires the following libraries (see the project page for the most up-to-date requirements):
  - ○ For basic CellProfiler Analyst functionality
    - ■ Python 2.8 or greater (3.0 is not currently supported)
    - ■ NumPy 1.71 or greater
    - ■ SciPy
    - ■ wxPython
    - ■ scikit-learn
    - ■ MySQLdb
    - ■ matplotlib
    - ■ javabridge
    - ■ python-bioformats
    - ■ verlib (required by distutils)
  - ○ For Tracer functionality
    - ■ Enthought Tool Suite (for Mayavi2)
    - ■ VTK, 5.10 or greater
    - ■ NetworkX, 1.7 or greater
    - ■ configobj (required by Enthought)
- **License:** GNU General Public License, Version 2.
- **Any restrictions to use by non-academics:** None

## Additional files

Additional file 1: Figures S1–S6. Referenced in the main manuscript text. (PDF 742 kb) Additional file 2:
**Details of the cellular image data including cell type, acquisition technique, resolution and temporal resolution.** (PDF 119 kb) Additional file 3:
**Listing of high-content image-based features generated by CellProfiler, categorized by feature type.** (XLS 33 kb)
