# Supplementary material for: CellProfiler Tracer: exploring and validating high-throughput, time-lapse microscopy image data
Source: BMC Bioinformatics. 2015 Nov 4;16:368. doi: 10.1186/s12859-015-0759-x (PMC4634901; doi:10.1186/s12859-015-0759-x)
Supplement: Additional file 1: Figures S1–S6. — Referenced in the main manuscript text. (PDF 742 kb) [file 12859_2015_759_MOESM1_ESM.pdf]

Additional Figures

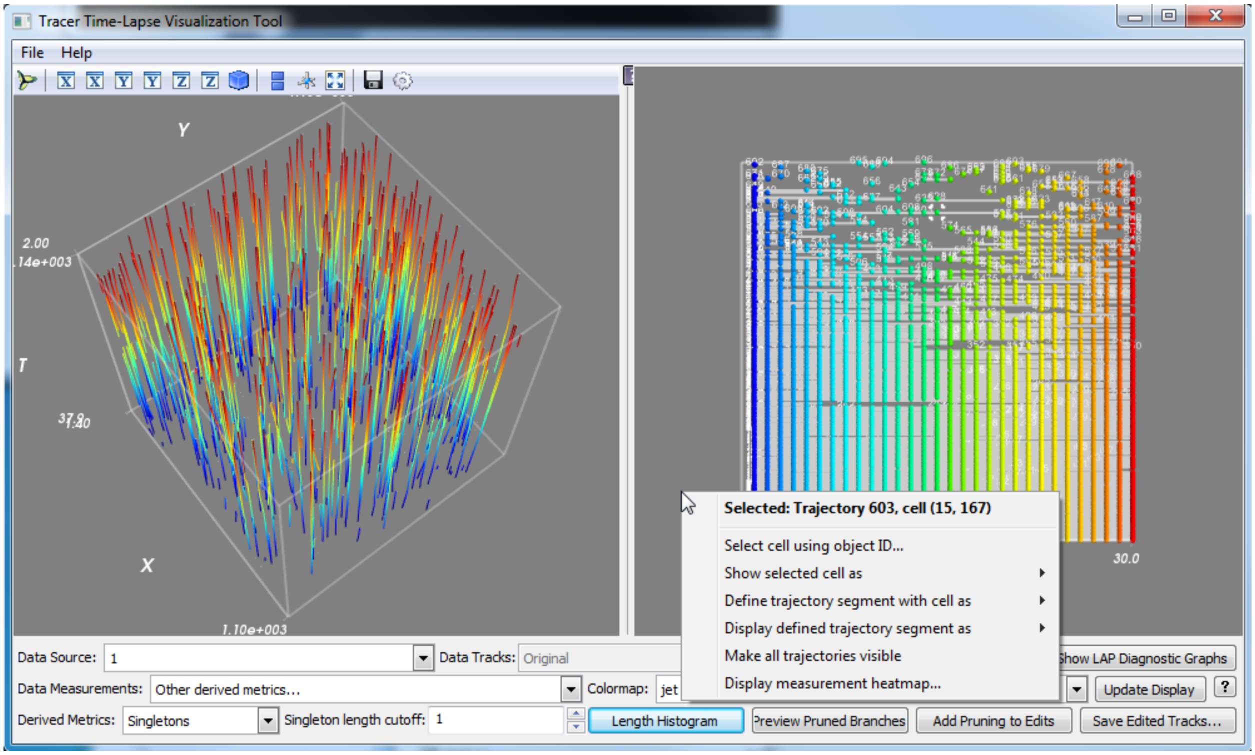

Additional Figure 1: A screenshot of the Tracer software displaying a data set containing hundreds of cells. This data set consists of 696 object tracks across 30 frames. The trajectory color can represent any collected cellular measurement; in the screenshot, the color represents the frame number. A right-click on either the XYT or lineage plots provides a context menu with options including trajectory selection, producing displays, plots and data tables of the collected per-cell image features. Details on various context-menu options are provided at the Tracer webpage at http://cellprofiler.org/tracer/.

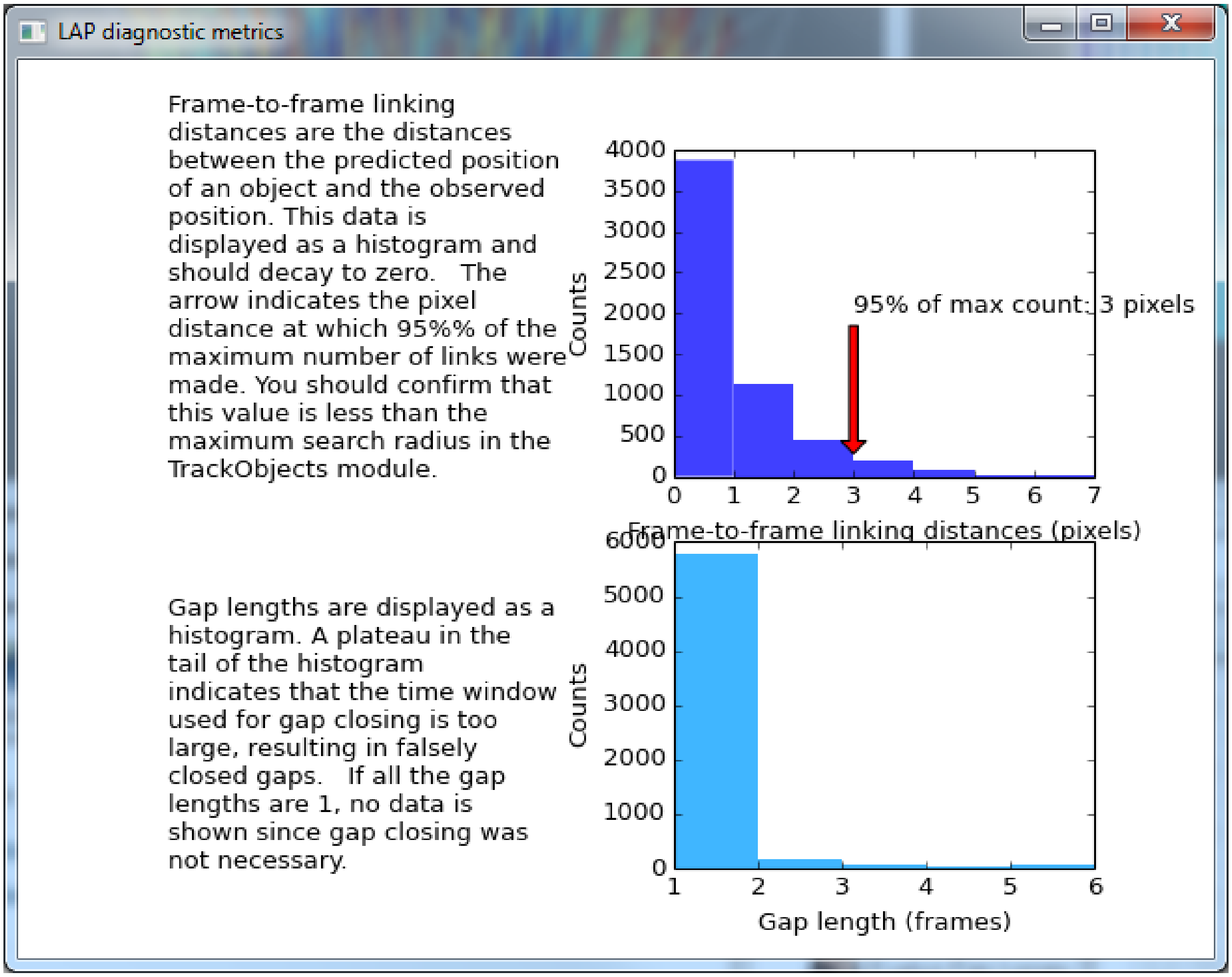

Additional Figure 2: A screenshot of the diagnostics associated with the LAP tracking algorithm. The LAP diagnostics are detailed in reference (Jaqaman and Danuser 2009). The top figure is a histogram of the frame-to-frame linking distances (measured in pixels), the bottom is a histogram of the trajectory gap lengths (measured in frames). Both plots are accompanied by explanatory text to aid in interpretation.

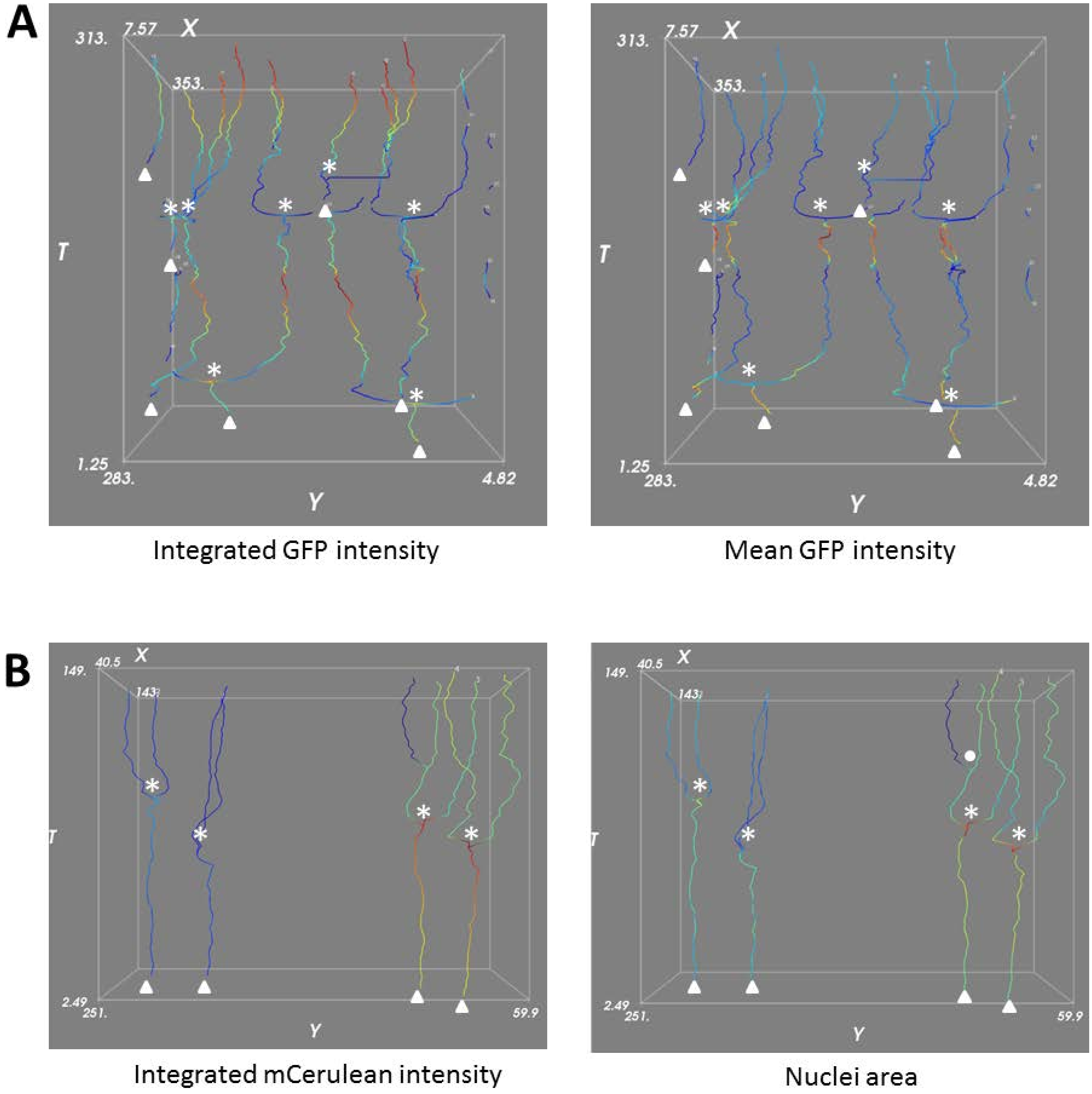

Additional Figure 3: Multi-parametric data displayed as color-coded trajectories in the Tracer XYT panel. Viewing cell measurement values in the context of object tracks can lead to biological interpretations. (A) *Drosophila* blastoderm embryo with GFP-histone marking the nuclear DNA. Nuclei divide in synchrony and characteristic changes in two particular measured features can be seen prior to and after cell division. (B) MCF-7 nuclei tagged with NLS-mCerulean fusion protein; here the measurements do not show dramatic and characteristic changes at cell divisions, largely due to the fact that the mammalian cells go out of focus at points during cell division. In all panels, arrows indicate objects at the starting timepoint and asterisks indicate a cellular division; for the right panel in (B), the filled circle indicates the formation of a micronucleus. These markings are not present in the actual software display but are shown here to better convey the 2D+*t* data in two dimensions, for publication.

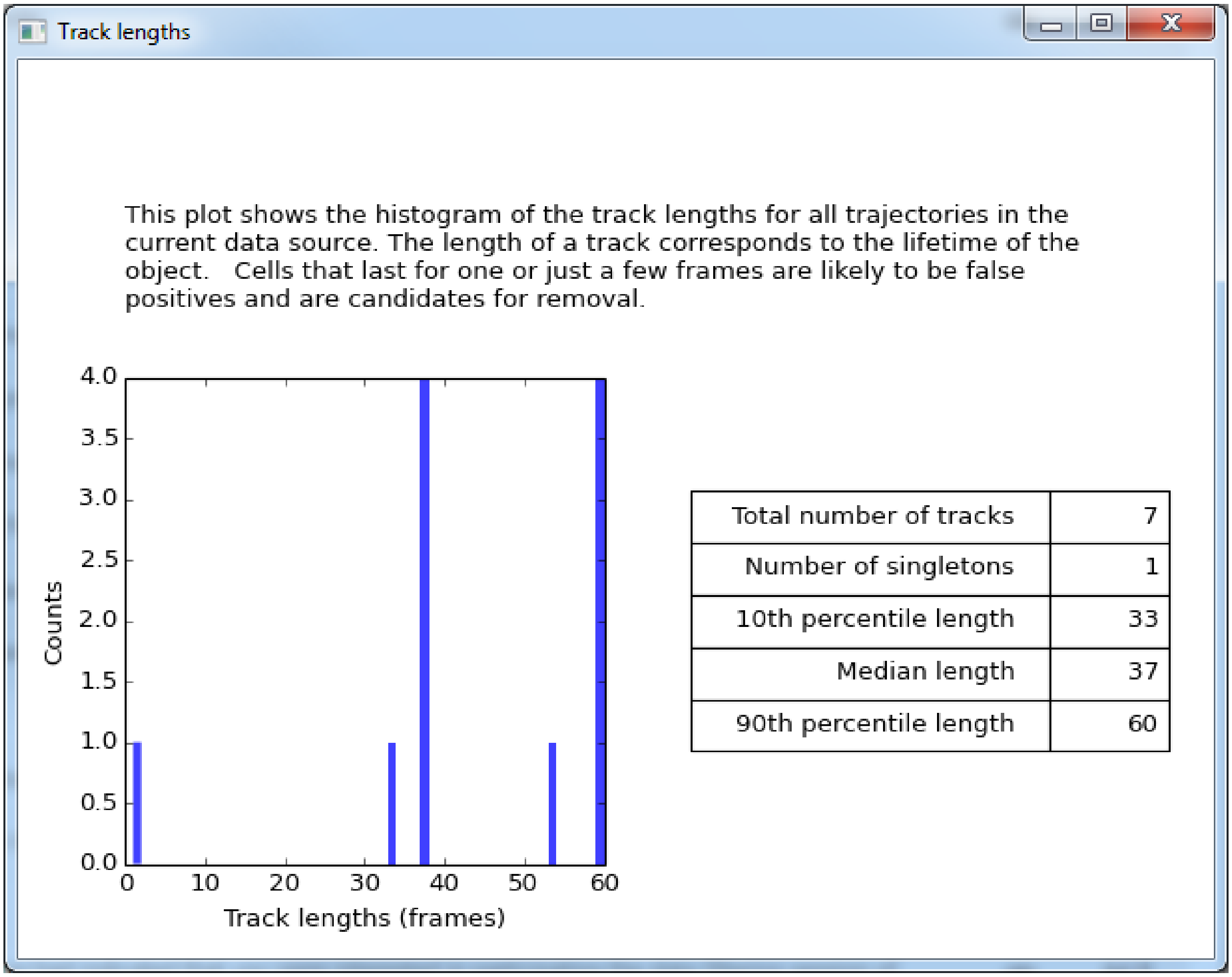

Additional Figure 4: A screenshot of the diagnostics associated with the “Singleton” tracking quality metric. The statistics are accompanied by explanatory text to aid in interpretation.

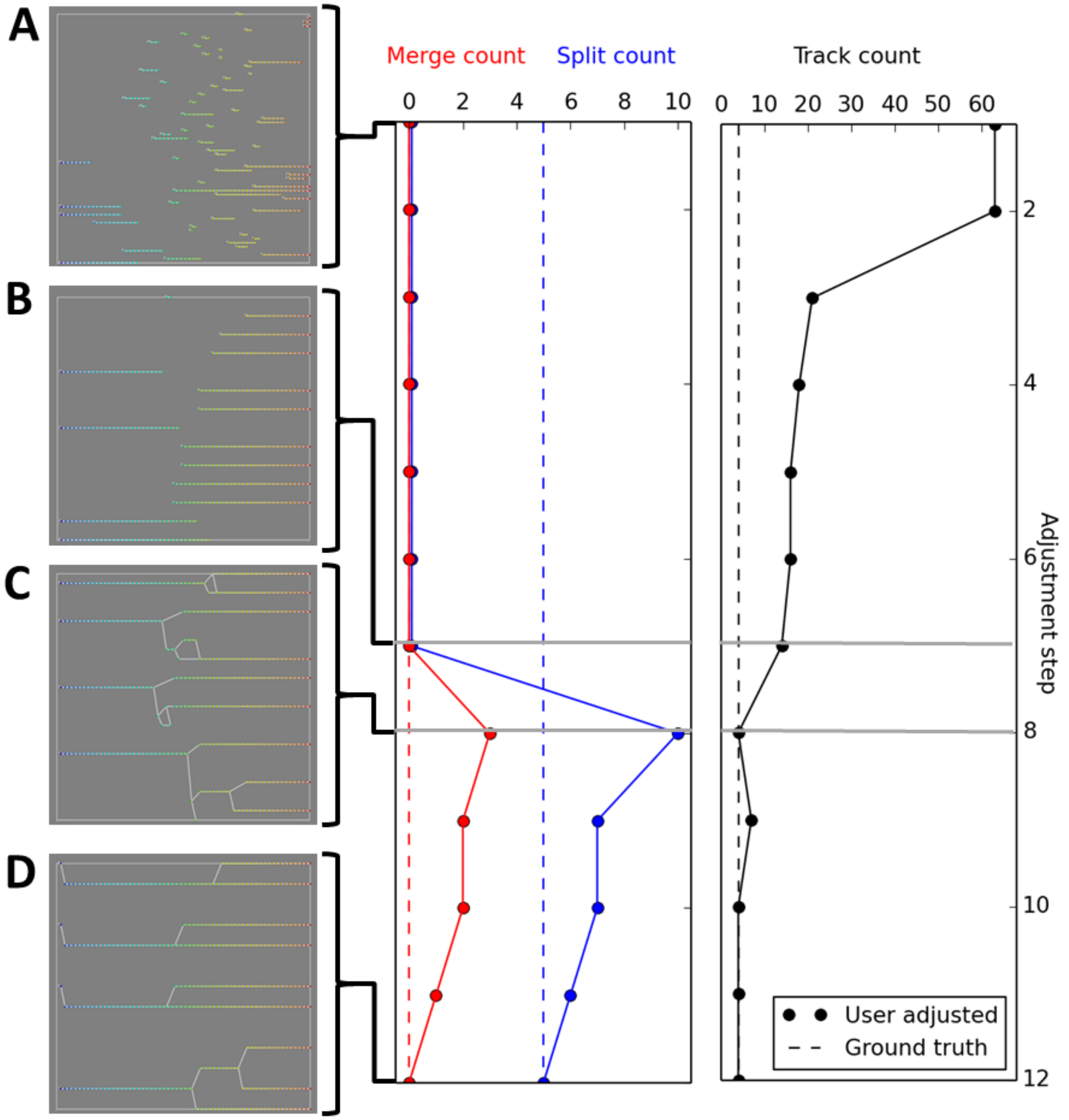

Additional Figure 5: Example use of Tracer for optimizing tracking algorithm parameters. The LAP tracking algorithm was used in CellProfiler's *TrackObjects* module to track MCF-7 cells across a sixty-frame movie. Tracer was used to visualize the tracking data and adjust the settings of that algorithm appropriately. The left-hand panels show the lineage panel view from Tracer; other visualizations and tools within Tracer were also used by the researcher to assess progress. The trajectory color-coding indicates the frame number. The middle panel charts the number of object splits (red) and merges (blue) detected in the full movie. The right-hand panel charts the number of tracks detected. For all charts, the correct (ground truth) value for the movie is shown as a dotted line. The plots are shown for four particular points in the optimization process: (A) At the beginning, using the LAP method's frame-to-frame linking step only, with default settings. (B) After the frame-to-frame linking step has been optimized. (C) Immediately thereafter, with the LAP method's gap closing step enabled, but with default settings. (D) At the end of all optimization.

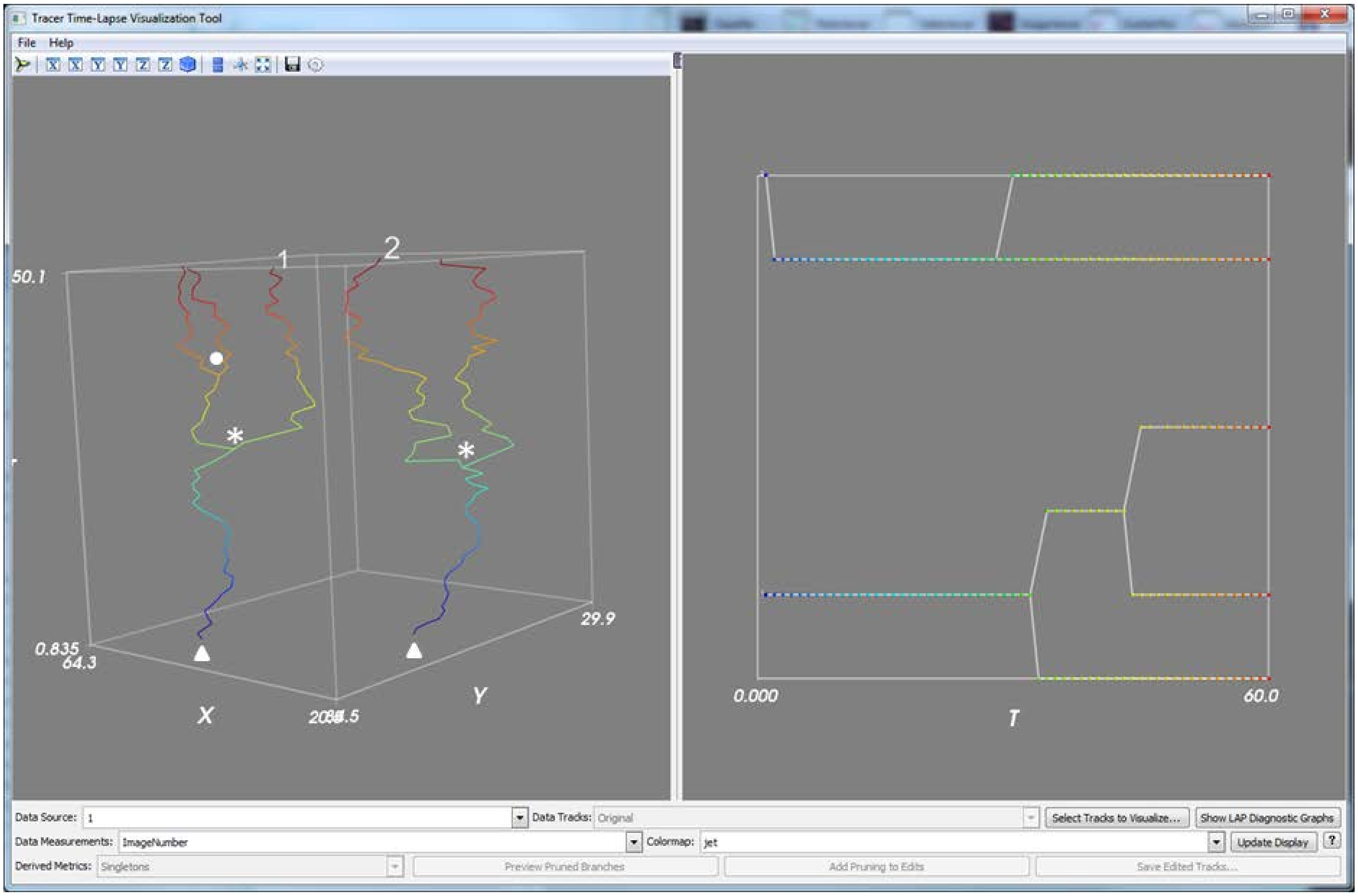

Additional Figure 6: Confirmation in Tracer of applying optimized tracking settings to another data set. The final settings used in CellProfiler's *TrackObjects* module to yield the tracking results shown in Additional Figure 5 were applied to a different MCF-7 data set; the same image acquisition and nuclei segmentation settings were used for both sets of data. The trajectory color-coding indicates the frame number. Arrows indicate objects at the starting timepoint, asterisks indicate a cellular division, the filled circle indicates the formation of a micronucleus. These markings are not present in the actual software display but are shown here to better convey the 2D+*t* data in two dimensions, for publication.
