# Supplementary material for: CellProfiler Tracer: exploring and validating high-throughput, time-lapse microscopy image data
Source: BMC Bioinformatics. 2015 Nov 4;16:368. doi: 10.1186/s12859-015-0759-x (PMC4634901; doi:10.1186/s12859-015-0759-x)
Supplement: Additional file 2: — Details of the cellular image data including cell type, acquisition technique, resolution and temporal resolution. (PDF 119 kb) [file 12859_2015_759_MOESM2_ESM.pdf]

Supplemental File: Time-lapse data details

MCF-7 image data

Acquired by: Albert Yeh, Laboratory of Sridhar Ramaswamy, Centers for Cancer Research, Regenerative Medicine, Human Genetics Research, and Systems Biology, Massachusetts General Hospital, Boston, MA 02114

Cell type: MCF-7 tagged with NLS-mCerulean fusion protein (NLS = nuclear localization signal), grown in DMEM

Acquisition technique: Serial imaging performed using an inverted microscope fitted in a standard-sized tissue culture incubator (Nikon Biostation CT)

Resolution: 10x magnification

Time interval between frames: 20 minutes

Drosophila embryo image data

Acquired by: Victoria Foe, Center for Cell Dynamics, Friday Harbor Labs, University of Washington, Friday Harbor, WA 98250

Cell type: A portion of a time lapse movie of a syncytial blastoderm stage *Drosophila* embryo with a GFP-histone gene which renders chromatin fluorescent in live embryos. The movie shows nuclear divisions 10 and 11.

Acquisition technique: Imaging performed on a Bio-Rad Radiance 2000 laser scanning confocal microscope using a 1.3NA oil objective.

Resolution: 40x magnification

Time interval between frames: 7 seconds
